# Effect of carbon source on lipase production by *Aeromonas *sp. isolated from dairy effluent

**DOI:** 10.1186/1753-6561-8-S4-P245

**Published:** 2014-10-01

**Authors:** Marcelo Rodrigues de Melo, Thaise Celini Moraes, Cristiani Baldo da Rocha, Fabiana Guillen Moreira Gasparin, Maria Antonia Pedrine Colabone Celligoi

**Affiliations:** 1 Departamento de Bioquímica e Biotecnologia, Universidade Estadual de Londrina, Londrina, PR, Brazil

## Background

Lipids are one of the major pollutants in domestic and industrial effluents. The use of lipases in the treatment of these effluents as well as in the bioremediation of contaminated environments represents an environmentally safe alternative to chemical methods [1]. Lipases (EC 3.1.1.3) are carboxylesterases that catalyze synthesis and hydrolysis of long-chain acylglycerols (>10 carbons) and have great potential for industrial and biotechnological applications. Microorganisms are the major source for lipases and have advantages such as ease production and diversified enzymatic properties [2]. Effluents containing high concentrations of lipids represent a good source for the isolation of lipase producing microorganisms and dairy industries are responsible for production of large quantities of this of this kind of effluent [3]. In a previous study two lipase producing microorganisms were isolated from dairy effluents. This report presents the results of lipase production by these microorganisms in different carbon sources.

## Methods

Isolates LODO 9 and LODO 10 were cultured overnight in DYG'S media (28°C, 200 rpm) and genomic DNA was extracted using AxyPrep™ Bacterial Genomic DNA Miniprep Kit (Axygen Biosciences) according to manufacturer recommendation. The 16S rDNA was amplified from chromosomal DNA using primers fD1 (AGAGTTTGATCCTGGCTCAG) and rD1 (AAGGAGGTGATCCAGCC) for *Escherichia coli *K-12 [4]. The 16S rRNA gene sequences obtained were compared with sequences of other *Aeromonas *deposited in the GenBank database by using ClustalW program and a consensus neighbor-joining tree was constructed using Molecular Evolutionary Genetics Analysis (MEGA) Software Version 4.0 [5]. Isolates were grown (30°C, 200 rpm, 48 h) in minimal medium (30 ml) containing (g/L): NaNO_3 _(4.0), KH_2_PO_4 _(1.5) FeCl_3 _(0.05), MgSO_4 _(0.2), CaCl_2 _(0.01), Na_2_HPO_4 _(0.5), yeast extract (0.05) and carbon source (10). Each carbon source (glucose, sucrose, lactose, cellobiose, xylose, glycerol, soybean oil, engine oil, diesel and gasoline) was tested separately from the other. The carbon source with best result was tested at concentrations of 0 to 250 g/L. The growth was accompanied by optical density at 600 nm and lipase activity by hydrolysis of *p*NPP (37°C, 10 min, 410 nm). A lipase unit (U) was defined as 1 mmol of pNP released per minute per ml supernatant under assay conditions.

## Results and conclusions

Isolates were identified as *Aeromonas *sp. (*Aeromonas *sp. LODO 9 and *Aeromonas *sp. LODO 10) and phylogenetic analysis grouped both isolates together, closely to *Aeromonas punctata *ATCC 15468. Lipase activity was detected only in cultures containing sucrose, cellobiose, glycerol, and soybean oil. The latter showed the best results with 95.02 and 87.80 U for *Aeromonas *sp. LODO 9 and *Aeromonas *sp. LODO 10, respectively. Both isolates reached the maximum lipase activity with 50 g/L of soybean oil. At this concentration of soybean oil, biomass and lipase activity of *Aeromonas *sp. LODO 9 were 6.38 mg/mL and 19.15 U, respectively. At this same condition, biomass and lipase activity of *Aeromonas *sp. LODO 10 were 8.55 mg/mL and 202.22 U, respectively.
